# Bovine Dermal Matrix as Coverage of Facial Nerve Grafts

**DOI:** 10.1155/2014/512921

**Published:** 2014-01-16

**Authors:** E. A. Kappos, P. E. Engels, R. Wettstein, D. J. Schaefer, D. F. Kalbermatten

**Affiliations:** Department of Plastic, Reconstructive, Aesthetic and Hand Surgery, University Hospital Basel, Spitalstraße 21, 4031 Basel, Switzerland

## Abstract

*Introduction*. Soft tissue defects over functional structures represent a challenge for the reconstructive surgeon. Often complex, reconstructive procedures are required. Occasionally, elderly or sick patients do not qualify for these extensive procedures. *Case*. We present the case of a 91-year-old lady with large hemifacial defect with exposed bone and nerves after tumor resection. We first performed radical resection including the fascia of the temporalis muscle and the frontal branch of the facial nerve. Due to the moribund elderly patient with a potentially high perioperative risk, we decided against flap reconstruction but to use bovine collagen/elastin matrix and split thickness skin graft. *Results*. No postoperative complications occurred and STSG and matrix healed uneventfully. *Discussion*. In selected cases, where complex reconstruction is not appropriate, this procedure can be a safe, easy, and fast alternative for covering soft tissue defects even on wound grounds containing nerve grafts.

## 1. Introduction

Soft tissue defects over functional structures represent a challenge for the reconstructive surgeon. Often complex, reconstructive procedures are required. Occasionally, elderly or sick patients do not qualify for these extensive procedures. In the case presented here, we used a new method of covering wide soft tissue facial defects with bovine collagen/elastin matrix (“bc/em”).

There are various ways to classify skin substitutes. One possibility was proposed by Halim et al. in 2010 [1] (for more details see Table 1).

We present the case of a 91-years-old lady with large defect with exposed bone and nerves after tumor resection. In order to decrease morbidity and time in hospital, we decided against flap reconstruction. The facial nerve had to be removed partially to guarantee in sano resection. While various cases using this method have been published [2–4], our case is unique in that it describes using the method on exposed upper facial nerve grafts.

## 2. Case

**Figure 1:**
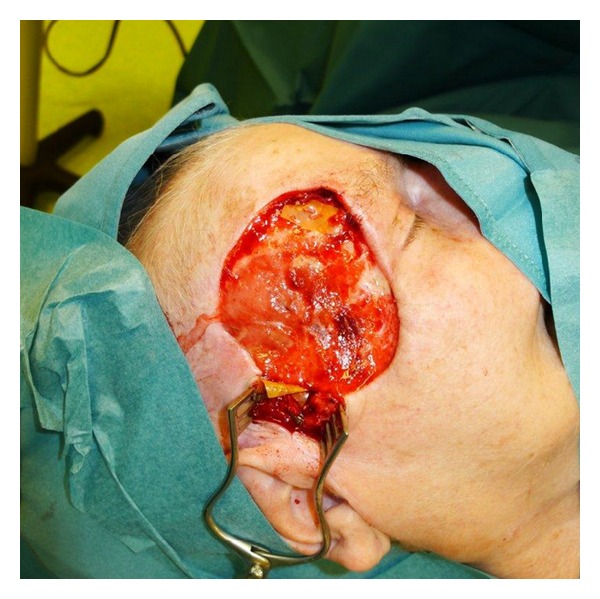
Defect after resection of tumor.

A 91 yo lady with type 2 diabetes, hypertension, and chronic cardiomyopathy presented with recurrence of sclerodermiform basalioma of right hemiface. MRI showed a subcutaneous tumor measuring 17 × 10 × 14 mm without signs of infiltration of fascia, muscle, or bone but facial nerve infiltration. We first performed radical resection including the fascia of the temporalis muscle and the frontal branch of facial nerve. A facial defect of 12 × 10 cm resulted with indication for nerve and soft tissue reconstruction (Figure 1).

Due to the moribund elderly patient with a potentially high perioperative risk, we decided against flap reconstruction but to use “bc/em” and STSG. Other substitutes like Matriderm would have been also a valuable option but in our case more expensive.

Surgery was performed under general anesthesia. First step surgery included facial nerve reconstruction. We used a cubital ulnar nerve graft measuring 7 cm.

**Figure 2:**
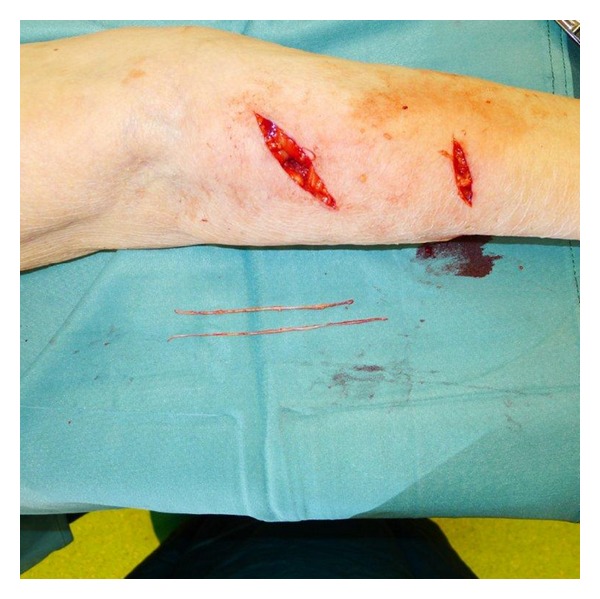
Nerve grafts.

Interfascicular splitting and microsurgical suturing to proximal and distal ending of facial nerve. Additional coverage with fibrin glue (Figure 2).

**Figure 3:**
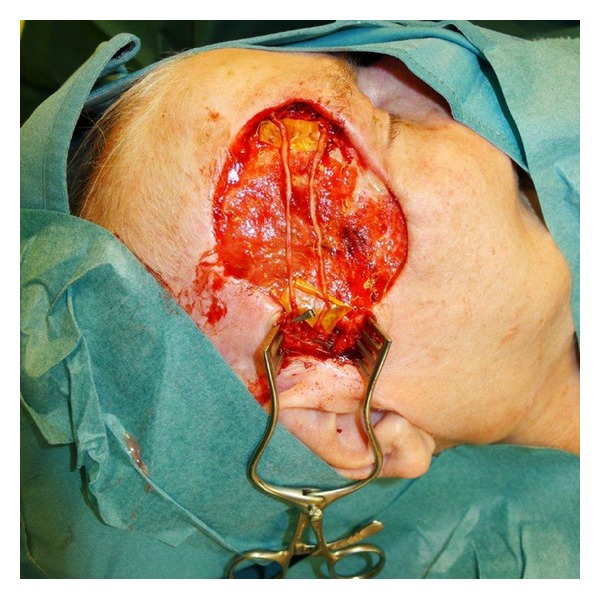
Nerve grafts after implantation.

**Figure 4:**
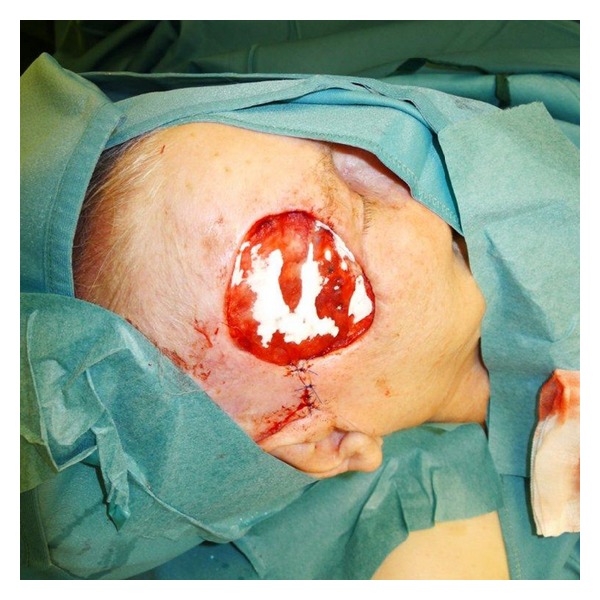
After implantation of the matrix.

**Figure 5:**
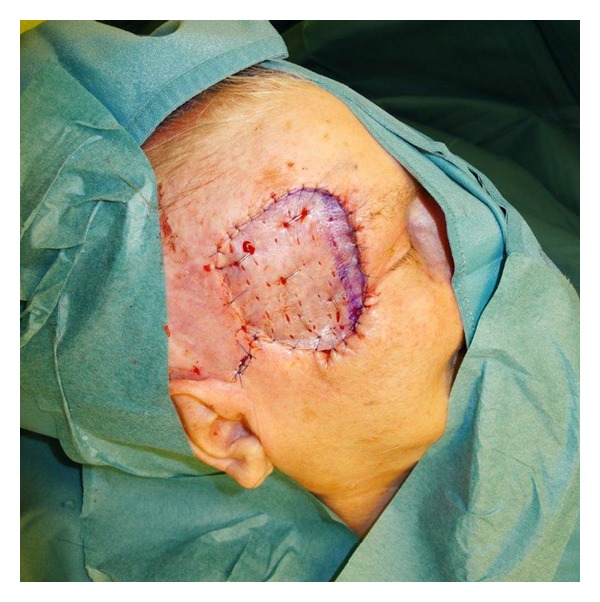
After implantation of skin graft.

**Figure 6:**
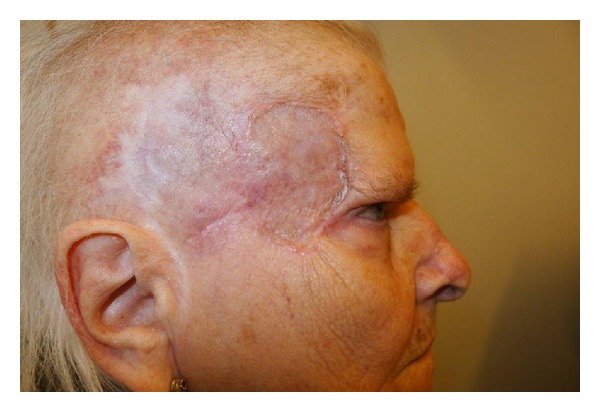
Result after 6 months.

The matrix was rehydrated in sterile saline solution and adjusted along the edges of the defect (Figure 3). Split thickness skin grafting (STSG) was added immediately, immobilizing the graft by suturing (Figures 4, 5, and 6).

## 3. Results

No postoperative complications occurred and STSG and matrix healed uneventfully. Six months after the surgery, we have so far only seen minimal active nerve recovery, but a static result that keeps the eyebrow in upper position. Furthermore, we classed the aesthetic result and soft tissue mobility in the reconstructed area as acceptable (Figure 6).

## 4. Discussion

Skin substitutes in general are a heterogeneous group of materials used for wound coverage that aid in wound closure and fulfill the functions of the skin, either temporarily or permanently, depending on the products characteristics [1]. They can be divided into two main groups [1, 5], biological and synthetic substitutes, which both have their advantages and disadvantages. The biological skin substitutes have a more intact extracellular matrix structure, while the synthetic skin substitutes can be synthesised on demand and can be modulated for specific purposes.

Biological skin substitutes allow for the development of a more natural new dermis and excellent reepithelialization characteristics due to the presence of a basement membrane. Synthetic skin substitutes have the advantage of increased control over scaffold composition. Looking back in history, the porcine wound model developed by De Vries et al. [6] in 1993 was the first successful animal model for the transplantation of a dermis/mesh transplant. In this model, matrices were covered with a split-skin mesh graft and protected with a microporous, semipermeable membrane to prevent blister formation and wound infection and to provide ideal healing conditions. This model allowed De Vries et al. to evaluate epithelization, dermal reconstitution, wound contraction, and cosmetic and functional aspects.

In an attempt to improve the scar quality, elastin was added in 1994 to the collagen matrix of the same animal model. A thicker dermis with less wound contractions due to reduced fibro- and myoblast invasion could be shown.

Middelkoop et al. [7] confirmed these results in vitro. Ascorbic acid was added to the native collagen-elastin construct leading to increased collagen synthesis. In 1995, it was again De Vries et al. [8] who used a human punch biopsy model to prove that native collagen matrices with elastin contribute to dermal regeneration and can reduce wound contraction.

Using a porcine wound model, a fastened neovascularisation in “bovine collagen/extracellular matrix” (“bc/em”) as compared to only STSG could be shown by Lamme et al. [9].

Haslik et al. [10] considered “bc/em” as a promising dermal substitute for the treatment of severe hand burns. Ryssel et al. [11] were able to show an equal healing rate and better skin elasticity of the “bc/em”/STSG transplants in burned victims after three months when compared to STSG only.

Over the years, various indications for “bc/em” were successfully tested; Keck and Ueberreiter [12] showed the successful correction of an adherent scar on the dorsal hand with “bc/em”, resulting in normal mobility between the skin and the underlying tissue and a consecutively fully regained range of motion.

A review of relevant literature points to the relatively late debridement of burn wounds as one possible reason for the lack of evidence of scar improvement after the use of “bc/em.” Midterm studies suggest that an early approach within the first few days after trauma may be preferable. An important advantage is the possible reduction of scar contraction and improved functionality of the involved areas.

As described by Heckmann et al. [13] in 2011, “bc/em”/STSG can not only be used to replace damaged or missing dermis in burned victims but also be used as a valuable flap alternative, even for the closure of large defects on bradytrophic ground with exposed tendon or bone (one possible explanation being the above-mentioned upregulated neovascularization in combination with STSG).

Haik and Zilinski recently published a review on 7 cases and found Matriderm especially useful in difficult areas where the desired result is a scar of the highest quality [14].

A wide variety of applications of “bc/em” have already been published, but to our knowledge there has been no report about its use in combination with nerve grafting.

We conclude that “bc/em” is a valuable alternative to “STSG only” grafts for wide, immediate dermis replacement. Indications vary from second and third degree burns [15–17] to décollement injuries and scar correction. Mesh on top of the “bc/em” matrix is an irreplaceable adjunct. Application is simple and the healing rate is comparable with “STSG only.”

The anticipated scar quality improvement, possibly objectified through elasticity measurements, has yet to be shown in clinical studies.

The above-mentioned case demonstrates that “bc/em”/STSG can be a valuable asset in plastic-reconstructive surgery. Depending on the case, one should consider combining different reconstructive approaches whenever possible. In the case discussed, a combination of “bc/em”, skin graft, nerve autograft, and local flap-reconstruction was used to minimise donor site morbidity. This one-step procedure may also reduce stress for the patient. A combination of these methods can lead to a functionally and aesthetically favourable result without the complexity and risks involved in a flap reconstruction for nonqualified patients.

There is a consensus among experienced surgeons that “bc/em”/STSG cannot replace flap reconstruction. Functionally, the latter is to be preferred due to its multilayer structure, weight-bearing capacity, and vascularisation. In selected cases, however, where complex reconstruction is not appropriate, “bc/em”/STSG can be a safe, easy, and fast alternative for covering soft tissue defects even on wound grounds containing a nerve graft. This relatively simple procedure satisfied the surgeons' and the patient's expectations offering a solution resulting in minimal morbidity to the elderly patient.

## Figures and Tables

**Table 1:** 

Temporary dressing materials	Single layer durable skin substitutes	Composite skin substitutes
(a) Single layer materials (i) Naturally occurring or biological dressing substitute, for example, amniotic membrane, potato peel (ii) Synthetic dressing substitute, for example, synthetic polymer sheet (Tegaderm, Opsite), polymer foam or spray	(a) Epidermal substitutes, for example, cultured epithelial autograft (CEA), Apligraft	(a) Skin graft (i) Allograft (ii) Xenograft

(b) Bilayered tissue engineered materials, for example, TransCyte	(i) Dermal substitutes (ii) Bovine collagen sheet, for example, Kollagen (iii) Porcine collagen sheet (iv) Bovine dermal matrix, for example, Matriderm (v) Human dermal matrix, for example, Alloderm	(b) Tissue engineered skin (i) Dermal regeneration template, for example, Integra (ii) Biobrane

## References

[1] Halim AS, Khoo TL, Mohd. Yussof SJ (2010). Biologic and synthetic skin substitutes: an overview. *Indian Journal of Plastic Surgery*.

[2] Golinski PA, Zöller N, Kippenberger S, Menke H, Bereiter-Hahn J, Bernd A (2009). Development of an engraftable skin equivalent based on Matriderm with human keratinocytes and fibroblasts. *Handchirurgie Mikrochirurgie Plastische Chirurgie*.

[3] van Zuijlen PM, van Trier AJM, Vloemans JFPM, Groenevelt F, Kreis RW, Middelkoop E (2000). Graft survival and effectiveness of dermal substitution in burns and reconstructive surgery in a one-stage grafting model. *Plastic and Reconstructive Surgery*.

[4] Lamy J, Gourari A, Atlan M, Zakine G (2013). Use of Matriderm 1 mm in reconstructive surgery. Series of 31 cases. *Annales de Chirurgie Plastique Esthétique*.

[5] Shakespeare P, Shakespeare V (2002). Survey: use of skin substitute materials in UK burn treatment centres. *Burns*.

[6] De Vries HJ, Mekkes JR, Middelkoop E (1993). Dermal substitutes for full-thickness wounds in a one-stage grafting model. *Wound Repair Regen*.

[7] Middelkoop E, de Vries HJC, Ruuls L, Everts V, Wildevuur CHR, Westerhof W (1995). Adherence, proliferation and collagen turnover by human fibroblasts seeded into different types of collagen sponges. *Cell and Tissue Research*.

[8] De Vries HJC, Zeegelaar JE, Middelkoop E (1995). Reduced wound contraction and scar formation in punch biopsy wounds. Native collagen dermal substitutes. A clinical study. *British Journal of Dermatology*.

[9] Lamme EN, de Vries HJC, van Veen H, Gabbiani G, Westerhof W, Middelkoop E (1996). Extracellular matrix characterization during healing of full-thickness wounds treated with a collagen/elastin dermal substitute shows improved skin regeneration in pigs. *Journal of Histochemistry and Cytochemistry*.

[10] Haslik W, Kamolz L-P, Nathschläger G, Andel H, Meissl G, Frey M (2007). First experiences with the collagen-elastin matrix Matriderm as a dermal substitute in severe burn injuries of the hand. *Burns*.

[11] Ryssel H, Gazyakan E, Germann G, Öhlbauer M (2008). The use of MatriDerm in early excision and simultaneous autologous skin grafting in burns—a pilot study. *Burns*.

[12] Keck M, Ueberreiter K (2008). Successful correction of an adherent scar on the dorsal hand with Matriderm. *Handchirurgie Mikrochirurgie Plastische Chirurgie*.

[13] Heckmann A, Radtke C, Rennekampff HO, Jokuszies A, Weyand B, Vogt PM (2012). One-stage defect closure of deperiosted bone and exposed tendons with MATRIDERM and skin transplantation—possibilities and limitations. *Der Unfallchirurg*.

[14] Haik J, Zilinski I (2012). Reconstruction of full-thickness defects with bovine-derived collagen/elastin matrix: a series of challenging cases and the first reported post-burn facial reconstruction. *Journal of Drugs in Dermatology *.

[15] Kolokythas P, Aust MC, Vogt PM, Paulsen F (2008). Dermal subsitute with the collagen-elastin matrix matriderm in burn injuries: a comprehensive review. *Handchirurgie Mikrochirurgie Plastische Chirurgie*.

[16] Wainwright D, Madden M, Luterman A (1996). Clinical evaluation of an acellular allograft dermal matrix in full-thickness burns. *Journal of Burn Care and Rehabilitation*.

[17] Gould D (1996). Endotracheal suctioning: an example of the problems of relevance and rigour in clinical research. *Journal of Clinical Nursing*.

